# T1 values in discrimination between health and disease using different T1 sequences: comparison between 3'3'5-MOLLI, 3'5-MOLLI, shMOLLI and SASHA

**DOI:** 10.1186/1532-429X-16-S1-P357

**Published:** 2014-01-16

**Authors:** Islam Z Mahmoud, Ciara Cummins, Zakir Hussain, Toby Rogers, Darius Dabir, Tobias Voigt, David M Higgins, Eike Nagel, Tobias Schaeffter, Valentina Puntmann

**Affiliations:** 1 King's College London, London, UK; 2 Philips Healthcare, Guilford, UK

## Background

Myocardial T1 mapping is emerging as a promising means to non-invasively discriminate between normal and diseased myocardium. Original modified Look-Locker sequence (3'3'5-MOLLI) can induce a long breath-hold and is prone to cardiac and respiratory motion. Several shorter sequences (3'5MOLLI, shMOLLI and SASHA) have been proposed for acquisition of T1 values. We examined native and postcontrast T1 values derived by above sequences within the same subject and investigated their ability to separate between health and disease. We further examined reproducibility of measurements within and between observers.

## Methods

Thirty-five consecutive subjects were enrolled in this study. Twenty-six healthy subjects served as controls. Single equatorial short-axis slice T1 mapping was performed using a 3-T scanner. T1 values were quantified within the septal, lateral myocardium and LV blood pool (native and post contrast). T1 relaxation maps were obtained using Relax-Maps tools supported by the PRIDE environment (Philips Healthcare). Inter and intra-observer variations were analysed. Statistical analysis was performed using SPSS.

## Results

**Figure 1:**
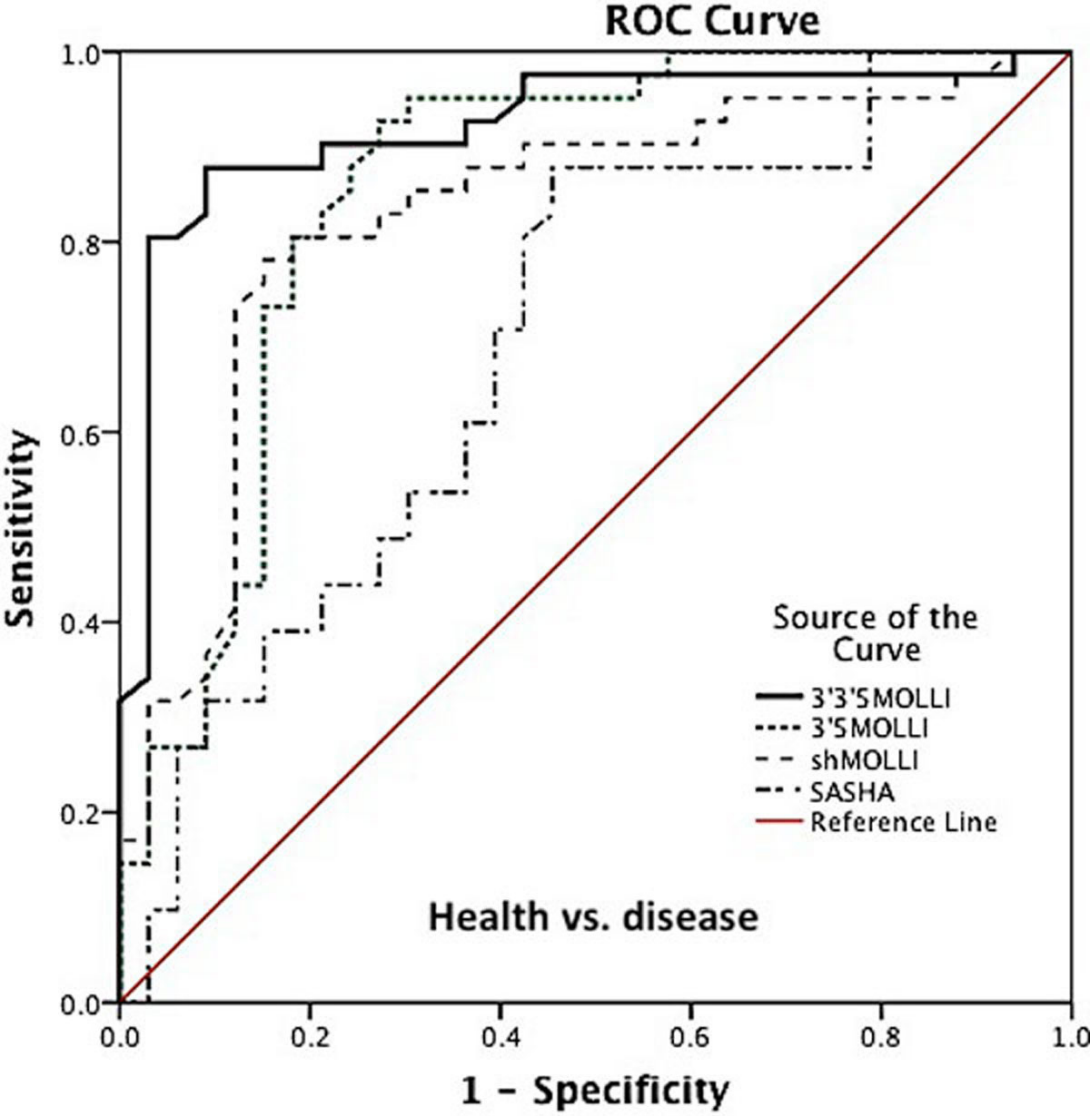


**Figure 2:**
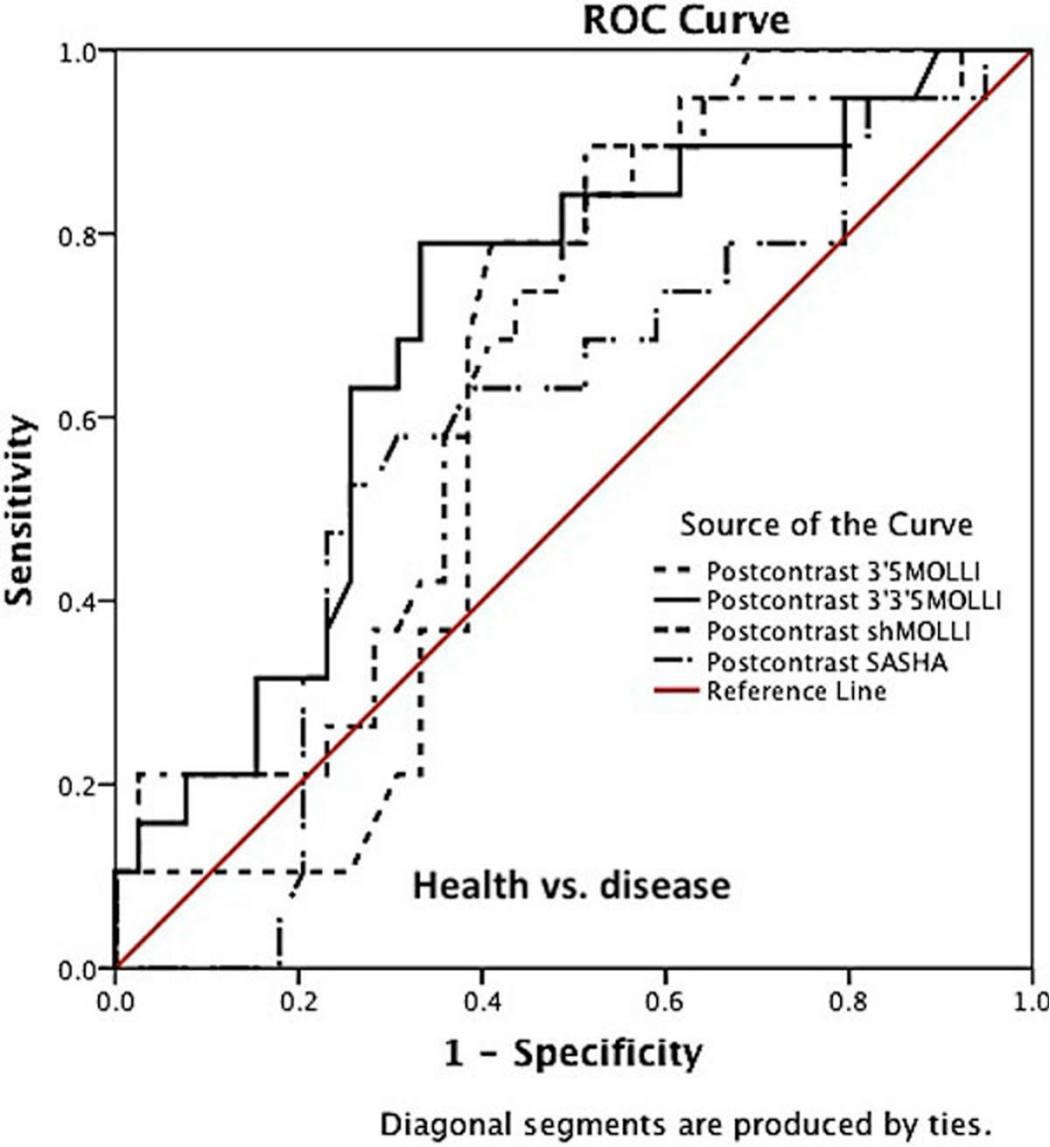


For native septal T1 imaging there was a significant difference between the health and disease using all sequences (Area under the curve, p-value: 3'3'5MOLLI vs 3'5MOLLI vs. shMOLLI vs SASHA: 0.92, p < 0.01 vs 0.89, p < 0.01, vs. 0.85, p < 0.01, 0.70, p < 0.04, Figure 1). In discrimination between ischaemic and nonischaemic cardiomyopathy, shMOLLI and SASHA could not discriminate against ischaemic cardiomyopathy. For postcontrast T1 imaging SASHA could not discriminate between health and disease (Figure 2). Interobserver mean differences for native septal T1 values for 3'3'5 MOLLI, 3'5 MOLLI, shMOLLI and SASHA were 1, 10, 10 and 16 (ms), respectively, whereas for post-contrast septal T1 values were 0.3, 3, 3 and 5 (ms), respectively. Intraobserver mean differences for native septal T1 values were 1, 1, 3 and 4 (ms), respectively, and post-contrast septal T1 values were 7, 10, 5 and 2 (ms), respectively.

## Conclusions

All sequences are able to discriminate between health and disease within the given sample size, where original 3'3'5 MOLLI retains superior discriminatory accuracy for native and postcontrast acquisition. 3'5MOLLI shows the nearest approximation to original 3'3'5 MOLLI values. 3'3'5 MOLLI shows superior inter and intra-observer agreement in compared to 3'5 MOLLI, shMOLLI and SASHA sequence.

## Funding

Department of Health via the National Institute for Health Research (NIHR) comprehensive Biomedical Research Centre award to Guy's & St Thomas' NHS Foundation Trust in partnership with King's College London and King's College Hospital National Health Service Foundation Trust. Dr Islam Mahmoud was supported by Ministry of science of Egypt.

